# Integrating computational methods guided the discovery of phytochemicals as potential Pin1 inhibitors for cancer: pharmacophore modeling, molecular docking, MM-GBSA calculations and molecular dynamics studies

**DOI:** 10.3389/fchem.2024.1339891

**Published:** 2024-01-22

**Authors:** Abdulrahim A. Alzain, Fatima A. Elbadwi, Tagyedeen H. Shoaib, Asmaa E. Sherif, Wadah Osman, Ahmed Ashour, Gamal A. Mohamed, Sabrin R. M. Ibrahim, Eun Joo Roh, Ahmed H. E. Hassan

**Affiliations:** ^1^ Department of Pharmaceutical Chemistry, Faculty of Pharmacy, University of Gezira, Gezira, Sudan; ^2^ Department of Pharmacognosy, Faculty of Pharmacy, Prince Sattam Bin Abdulaziz University, Al-Kharj, Saudi Arabia; ^3^ Department of Pharmacognosy, Faculty of Pharmacy, Mansoura University, Mansoura, Egypt; ^4^ Department of Pharmacognosy, Faculty of Pharmacy, University of Khartoum, Khartoum, Sudan; ^5^ Department of Natural Products and Alternative Medicine, Faculty of Pharmacy, King Abdulaziz University, Jeddah, Saudi Arabia; ^6^ Preparatory Year Program, Department of Chemistry, Batterjee Medical College, Jeddah, Saudi Arabia; ^7^ Department of Pharmacognosy, Faculty of Pharmacy, Assiut University, Assiut, Egypt; ^8^ Chemical and Biological Integrative Research Center, Korea Institute of Science and Technology (KIST), Seoul, Republic of Korea; ^9^ Division of Bio-Medical Science and Technology, University of Science and Technology, Daejeon, Republic of Korea; ^10^ Department of Medicinal Chemistry, Faculty of Pharmacy, Mansoura University, Mansoura, Egypt

**Keywords:** Pin1, cancer, pharmacophore modeling, molecular docking, MM-GBSA, molecular dynamics

## Abstract

Pin1 is a pivotal player in interactions with a diverse array of phosphorylated proteins closely linked to critical processes such as carcinogenesis and tumor suppression. Its axial role in cancer initiation and progression, coupled with its overexpression and activation in various cancers render it a potential candidate for the development of targeted therapeutics. While several known Pin1 inhibitors possess favorable enzymatic profiles, their cellular efficacy often falls short. Consequently, the pursuit of novel Pin1 inhibitors has gained considerable attention in the field of medicinal chemistry. In this study, we employed the Phase tool from Schrödinger to construct a structure-based pharmacophore model. Subsequently, 449,008 natural products (NPs) from the SN3 database underwent screening to identify compounds sharing pharmacophoric features with the native ligand. This resulted in 650 compounds, which then underwent molecular docking and binding free energy calculations. Among them, SN0021307, SN0449787 and SN0079231 showed better docking scores with values of −9.891, −7.579 and −7.097 kcal/mol, respectively than the reference compound (−6.064 kcal/mol). Also, SN0021307, SN0449787 and SN0079231 exhibited lower free binding energies (−57.12, −49.81 and −46.05 kcal/mol, respectively) than the reference ligand (−37.75 kcal/mol). Based on these studies, SN0021307, SN0449787, and SN0079231 showed better binding affinity that the reference compound. Further the validation of these findings, molecular dynamics simulations confirmed the stability of the ligand-receptor complex for 100 ns with RMSD ranging from 0.6 to 1.8 Å. Based on these promising results, these three phytochemicals emerge as promising lead compounds warranting comprehensive biological screening in future investigations. These compounds hold great potential for further exploration regarding their efficacy and safety as Pin1 inhibitors, which could usher in new avenues for combating cancer.

## 1 Introduction

Cancer represents a formidable global health challenge, manifesting with millions of new incidences and fatalities reported annually. Projections forecast a persistent upward trend in cancer incidence, with an estimated annual caseload of nearly 22 million new instances expected by 2030 (Bray et al., 2015a; Bray et al., 2015b). The escalating economic burden stemming from cancer management is experienced globally, necessitating the exploration of novel, efficacious anticancer therapeutics (Zhang et al., 2023). While numerous genes have been implicated in tumorigenesis, only a fraction of these genes present druggable targets (Futreal et al., 2004; Malumbres and Barbacid, 2007). Molecularly targeted therapy stands as a promising approach to cancer treatment, as it holds the potential to mitigate the adverse effects associated with traditional chemotherapy. Nevertheless, targeting a solitary molecular pathway may fall short, given that cancer cells frequently employ alternative survival and proliferation mechanisms. In this context, the inhibition of proteins that govern multiple oncogenic pathways may prove to be a viable solution (Ciarcia et al., 2013).

One such protein exerting a pivotal role in regulating cellular pathways is Pin1. Pin1 functions as a catalyst for the conformational alteration of proline peptide bonds within Ser/Thr-Pro motifs, which serve as critical target sequences for kinases and phosphatases (Lu et al., 1996). The isomerization of these motifs exerts influence over the phosphorylation status of numerous proteins, thereby affecting their functions and stability (Lu et al., 2007; Russo Spena et al., 2018). Pin1 plays a central role in various biological processes, including the cell cycle, proliferation, motility, cellular survival, and apoptosis (Lu et al., 1996; Ranganathan et al., 1997; Pastorino et al., 2006). Dysregulation of Pin1 has been implicated in various pathological conditions, particularly cancer. It is typically found at low levels in normal tissues and cell lines, with fluctuations throughout the cell cycle. However, in several human malignancies, including ovarian, breast, prostate, gastric, lung, melanoma, and cervical cancers, Pin1 experiences overexpression and activation (Yu et al., 2020). Elevated Pin1 levels often correlate with unfavorable clinical outcomes, underscoring its potential prognostic value in cancer (Ayala et al., 2003; Fukuchi et al., 2006).

Considering that uncontrolled cell proliferation is a common hallmark of cancer, the inhibition of Pin1 holds the potential to simultaneously target multiple oncogenic signaling pathways at distinct levels (Wulf et al., 2005). Existing research indicates that Pin1 upregulates over 50 oncogenes while inhibiting more than 20 tumor suppressors (Chen et al., 2018). These attributes position Pin1 inhibitors as valuable assets in the battle against cancer and strong candidates for targeted therapy (Russo Spena et al., 2019). Several potent antagonists of Pin1, encompassing chemical compounds, peptide-based drugs, and nanoparticles, have been developed (Urusova et al., 2011; Moore and Potter, 2013). Notable examples include the NP Juglone and the small molecule PiB and its derivatives. However, these compounds display relatively modest inhibitory activity against Pin1 (Hennig et al., 1998; Uchida et al., 2003). Although nanomolar peptidic Pin1 inhibitors have been identified, their limited cell membrane permeability restricts their applicability (Wildemann et al., 2006). Existing inhibitors often fall short of the necessary levels of specificity, efficacy, and safety profiles for clinical utilization. Consequently, there is an imperative need for continued research and development endeavors to discover Pin1 inhibitors and amplify their therapeutic potential.

The interest in screening NPs for drug discovery and development is readily apparent in the scientific literature (Yan et al., 2014). A substantial proportion of drugs approved by the United States Food and Drug Administration (FDA) find their origins in plants or their derivatives (Patridge et al., 2016). Furthermore, more than 60% of drugs employed in the treatment of cancer consist of natural compounds and their derivatives (Carocho and Ferreira, 2013). NPs have garnered attention for several compelling reasons, including their relatively cost-effective production, minimal adverse side effects, and high degree of tolerability within the human body (Vahedi et al., 2008).

The pursuit of novel drugs is a resource-intensive and time-consuming endeavor (Leelananda and Lindert, 2016; Vemula et al., 2023). To streamline this process, integrating computer-based approaches during the initial stages of drug discovery has proven invaluable. One such strategy is Computer-Aided Drug Design (CADD) (Bray et al., 2015a; Macalino et al., 2015), which plays a pivotal role in identifying promising drug candidates through virtual screening. CADD not only aids in the optimization of candidate properties to maximize effectiveness and safety but also possesses the capability to generate entirely new compounds by combining distinct chemical fragments (Kapetanovic, 2008). Within drug discovery, CADD serves three essential functions: narrowing down a vast pool of compounds for experimental testing, refining the properties of potential drugs to ensure safety and efficacy, and facilitating the design of novel compounds by assembling different chemical building blocks (Wang et al., 2021). Molecular docking stands out as a widely utilized technique in virtual screening, particularly when the 3D structure of a protein is known (Prieto-Martínez et al., 2019). Three primary docking approaches—ensemble docking, induced fit docking, and lock and key docking—exist. Further categorization includes rigid ligand and rigid receptor docking, flexible ligand and rigid receptor docking, and flexible ligand and flexible receptor docking (Pagadala et al., 2017). Numerous software programs, available as both in-house tools and open-source applications, employ various algorithms and functions for the docking process.

In the exploration of the flexibility and dynamics of drug-target interactions, molecular dynamics (MD) simulations have emerged as a critical tool, revolutionizing the field of drug development and gaining widespread acceptance in computational methods such as CADD (Fukuchi et al., 2006). MD simulations facilitate detailed tracking of the movements and arrangements of individual particles, offering a comprehensive understanding of the microscopic-scale behavior of these tiny particles (Wulf et al., 2005).

This study encompassed pharmacophore modeling and screening, molecular docking, Molecular Mechanics-generalized Born surface area (MM-GBSA) calculations, and molecular dynamics (MD) simulations to identify structurally novel Pin1 inhibitors.

## 2 Materials and methods

All computational studies were carried out using Maestro v 12.8 of Schrödinger. Academic Desmond v6.5 by D.E. Shaw Research was used for MD.

### 2.1 Preparation of protein

The three-dimensional structure of Pin1 (chain B), identified by its PDB accession code 3I6C, was obtained from the Protein Data Bank (PDB). This structural data was resolved at a high resolution of 1.3 Å. In order to prepare the protein for rigorous analysis, the PPW (Protein Preparation Wizard) tool, an automated software component within the Schrödinger suite (Madhavi Sastry et al., 2013), was employed. The PPW tool executed a series of pivotal operations designed to rectify any inherent structural aberrations within the protein. The initial step encompassed preprocessing the protein, involving the elimination of water molecules residing within a radius of 5.0 Å from the protein’s structure. Subsequently, appropriate assignments of bond orders were executed, and heteroatoms that exerted negligible influence on the protein’s conformation were deleted. Hydrogen atoms were then systematically introduced to the carbon atoms within the protein structure. This concerted effort aimed at establishing the necessary chemical bonds essential for a realistic representation of the protein’s atomic composition. For the refinement and optimization of the protein’s spatial arrangement, the OPLS4 force field, a robust computational model for biomolecular simulations (Jorgensen and Tirado-Rives, 1988), was judiciously employed. This computational approach significantly enhanced the energetics of the protein, ensuring its stability and reliability for subsequent analysis. To further enhance the protein’s structural integrity and ensure its suitability for subsequent analyses, a restrained minimization procedure was performed, with the ultimate goal of achieving a Root Mean Square Deviation (RMSD) value of 0.30 Å. This final step was paramount in fine-tuning the protein’s conformation to an optimal state, paving the way for comprehensive and insightful investigation.

### 2.2 Retrieval of the database and grid generation

A library comprising 444,450 NPs was obtained from the SuperNatural 3.0 (SN3) database (https://bioinfapplied.charite.de/supernatural_3/subpages/compounds.php) for this study. These molecules underwent a rigorous minimization process using the Macromodel program, a widely accepted tool for molecular simulations and optimization in the scientific community. The application of the OPLS4 force field, renowned for its good accuracy in depicting protein-ligand interactions, was an integral part of this minimization procedure. To facilitate precise and reliable docking studies, the generation of a receptor grid was essential. The receptor grid generation task was accomplished using the default procedures embedded within the Glide module (Halgren et al., 2004). This entailed the careful selection of coordinates associated with a ligand that had previously formed a complex with the protein of interest. This ligand was employed as a reference point to construct a three-dimensional grid with dimensions, precisely mirroring the active binding site of the receptor.

### 2.3 E-pharmacophore generation

Structure-based pharmacophore design represents an approach for the systematic generation of a pharmacophore model, grounded in the structural characteristics of the protein target and the bioactive conformation of a co-crystallized ligand. This methodology is predicated upon the robust and unchanging nature of the target protein’s active site, as well as the specific conformational attributes and binding interactions of the ligand, which are elucidated through X-ray crystallographic structure analysis (Rella et al., 2006).

In this particular investigation, the Phase module, an integral component of Schrödinger’s software suite (Salam et al., 2009), was used to execute the pharmacophore modeling process. To construct the pharmacophore model, the receptor-ligand complex was employed as the foundation, utilizing the energy-based method (E-Pharmacophore) with the primary objective of incorporating a minimum of 5–6 features into the pharmacophore model. Notably, Phase employs a standardized set of six chemical features, encompassing hydrogen bond acceptor (A), hydrogen bond donor (D), positive ionizable (P), negative ionizable (N), hydrophobic (H), and aromatic ring (R) motifs, as the basis for generating the pharmacophore hypothesis (Dixon et al., 2006).

Throughout the hypothesis generation process, pharmacophoric sites were constructed around the atoms contributing to the overall energetic profile. The energy contributions of individual atoms within each pharmacophoric site were summated, and the sites were subsequently ranked based on their respective energy values (Salam et al., 2009). Ultimately, the resulting pharmacophore hypothesis was deployed to search the database, identifying compounds with superior potency compared to the reference ligand, thereby aiding in the selection of potential lead compounds for further exploration.

### 2.4 Pharmacophore-based ligand screening

The pharmacophore model, denoted as CPH, which was successfully generated, played a pivotal role in the screening of the SN3 database for potential inhibitors of Pin1. A comprehensive analysis encompassed the examination of all molecules contained within the database, with the objective of identifying compounds that exhibited full matches with all the constituent sites of the pharmacophore model.

### 2.5 Molecular docking and binding free energy calculations

Docking studies were executed employing the Glide software integrated within the Schrödinger interface (Halgren et al., 2004). Glide is a widely recognized and extensively used molecular docking tool for its reasonably accurate estimation of protein-ligand binding modes.

Glide incorporates two distinct scoring functions: SP (Standard Precision) and XP (Extra Precision). The two filters vary in terms of speed, accuracy, and scoring methodology. Specifically, the SP mode, with a screening rate of 20 s per compound, facilitates rapid compound screening by minimizing intermediate conformations, final torsion refinement, and sampling. In contrast, the XP mode adopts a more time-intensive approach, taking 2 min per compound, employing extensive sampling, and utilizing a sophisticated scoring function. This approach is designed to eliminate false positive compounds. Additionally, XP mode penalizes compounds exhibiting reduced form complementarity with the target active site (Friesner et al., 2004). In this study, the docking was performed in two successive steps, SP docking followed by XP docking.

The ranking of molecules was carried out based on their docking scores. To gain deeper insights into the protein-ligand interactions and to guide subsequent analysis, the ligand-interaction diagram tool in Maestro was employed.

Furthermore, it is noteworthy that recent reports emphasize the advantages of ranking inhibitors based on more precise binding free energy calculations, such as Molecular Mechanics/Generalized Born Surface Area (MM/GBSA), as opposed to relying solely on XP scores (Alzain et al., 2022). Consequently, for the most promising hits identified, molecular MM/GBSA calculations were executed using the Prime module within the Schrödinger interface. This energy estimation procedure featured the utilization of the OPLS4 force field for EMM (Energy Minimization Method) and SGBM (Surface Generalized Born Model) for VSGB (Volume Surface Generalized Born) calculations (Elbadwi et al., 2021).

### 2.6 Molecular dynamics simulation

To study the stability of the Pin1 receptor complexed with the top three molecules, Molecular Dynamics Simulations (MDS) were conducted utilizing the Desmond software (Ash and Fourches, 2017; Elbadwi et al., 2021; Alzain et al., 2022). Initially, the system was prepared by solvating it with a TIP3P water model within an orthorhombic periodic boundary box. The dimensions of the box were uniformly set to 10 Å for each axis, and the angles of the box were fixed at 90°. To maintain charge neutrality, chloride ions (Cl-) were introduced into the system, considering the overall charge of the model. Furthermore, a salt concentration of 0.15 M was incorporated to simulate physiological conditions. The model generated in the previous steps underwent energy minimization utilizing the Desmond minimization application. A maximum of 1,000 iterations were allowed, with convergence criteria set at 1.0 kcal/mol. All other relevant parameters were retained at their default values. The Smooth Particle Mesh Ewald (SPME) technique was deployed for handling long-range electrostatic interactions, employing a tolerance of 1e-09. Short-range electrostatic interactions were handled using a cutoff radius of 9 Å. Subsequently, the minimized model was employed for MDS within the NPT ensemble. These simulations were conducted at a temperature of 300 K and a pressure of 1 bar. The simulations extended over a duration of 100 nanoseconds (ns), employing the general sampling method.

A comprehensive analysis was conducted utilizing the trajectory file obtained from the molecular dynamics simulations. This analysis encompassed a range of assessments and evaluations to elucidate the behavior and interactions of the system under investigation.

## 3 Results and discussion

**FIGURE 1:**
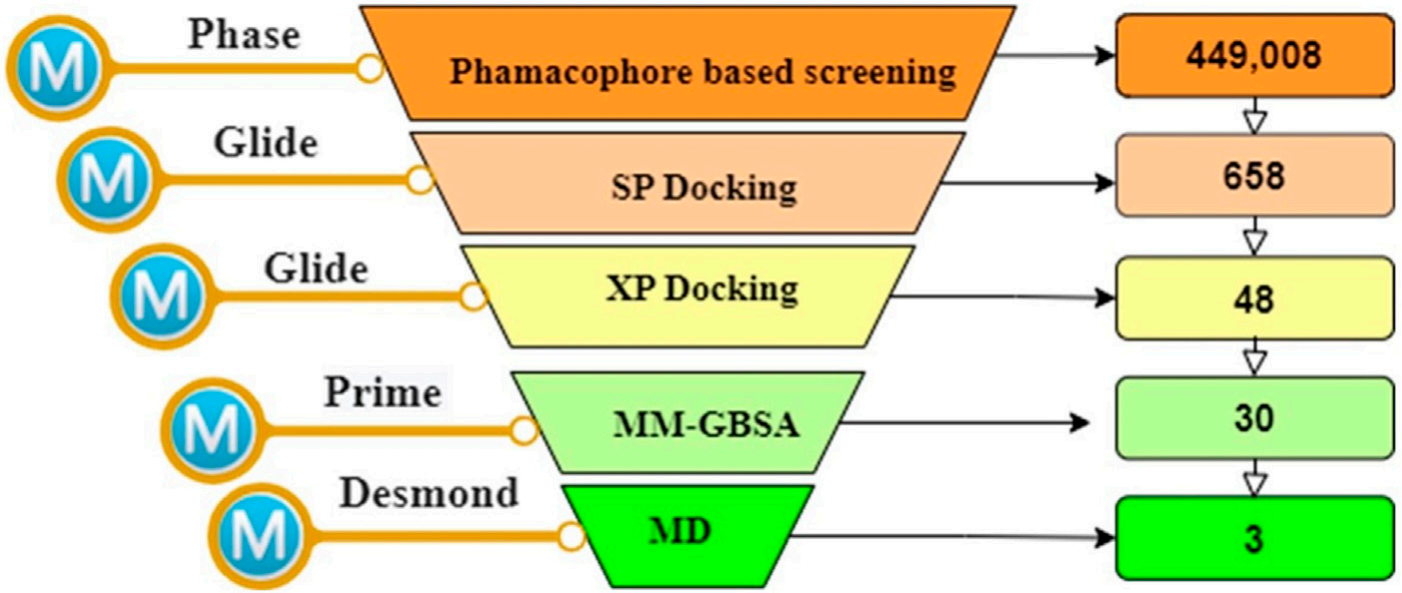
The overall works of the study.

The workflow of this study is summarized in Figure 1.

### 3.1 E-pharmacophore model and pharmacophore-based ligand screening

**FIGURE 2:**
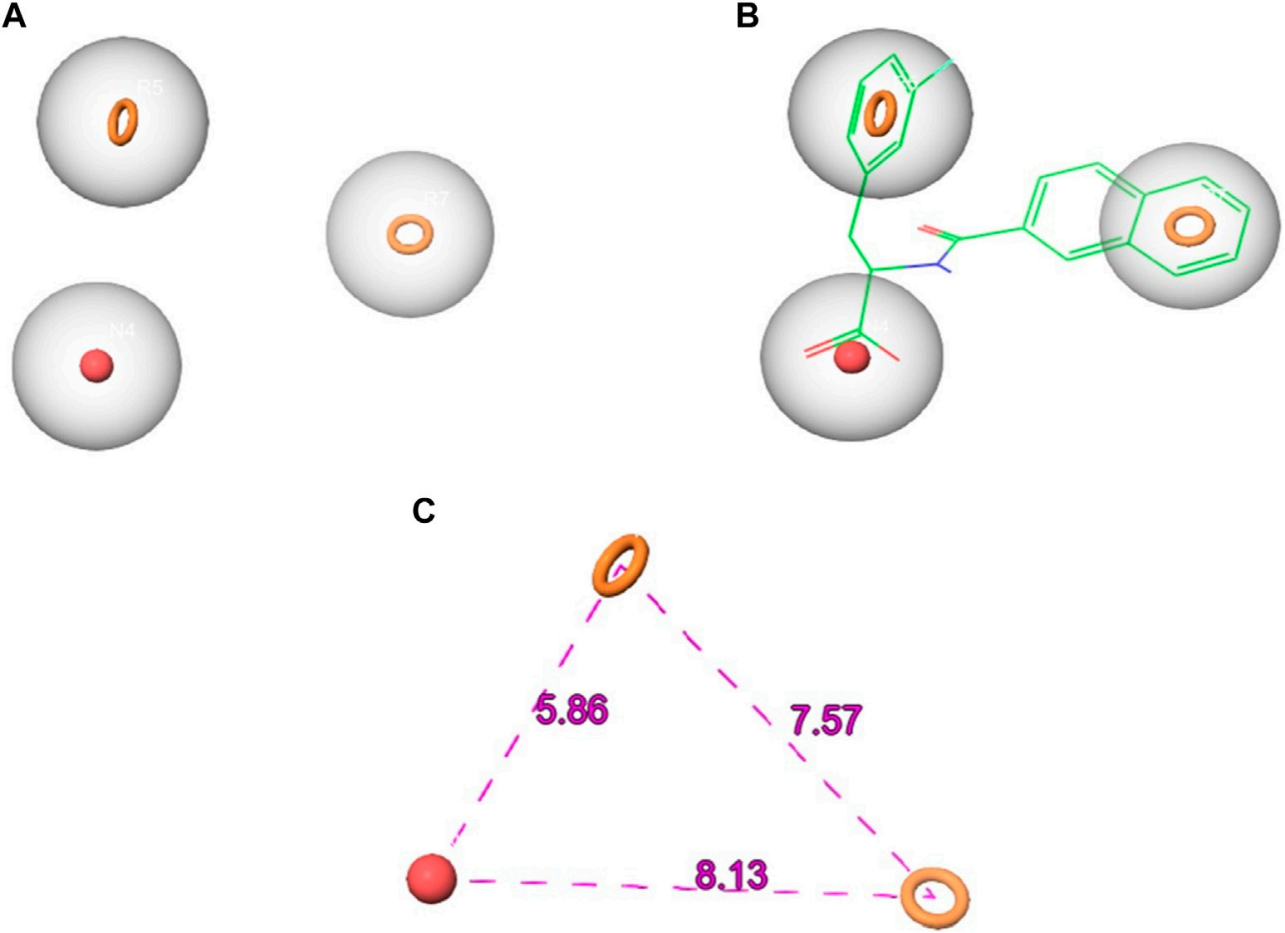
The generated pharmacophore hypothesis of Pin1 complexed with the reference **(A)** the hypothesis **(B)** hypothesis aligned with the reference ligand **(C)** hypothesis distance.

The Phase module was employed to construct an electronic pharmacophore (e-pharmacophore) model. This model was developed using the ligand bound to Pin1 and incorporated both structural and energetic attributes. The primary objective of the e-pharmacophore model was to discern favorable regions within the active site for the purpose of designing innovative inhibitors (Tuccinardi et al., 2016). In the course of e-pharmacophore generation, Phase retrieved three distinct pharmacophoric features. These features were used to generate a three-point e-pharmacophore model, comprising one negative ionizable (N) feature and two aromatic rings (R) features, denoted as NRR (Figure 2). The N4 feature exhibited a radius of 2 Å and an energy of −1.25 kcal/mol, while the R5 and R7 sites shared the same radius as N4 but had energies of −0.51 and −0.68 kcal/mol, respectively.

Subsequently, the e-pharmacophore model was employed to screen the SN3 database, aiming to identify potential Pin1 inhibitors. During the screening process, conformers of each ligand within the database were scrutinized to identify molecules that conformed to the e-pharmacophore model. The molecular sites were predefined to match the three-point e-pharmacophore model. The utilization of the e-pharmacophore model in screening led to the retrieval of 658 hit molecules that matched the three pharmacophoric features (NRR) from a total of 449,005 NPs in the SN3 database. It can be inferred that, given the e-pharmacophore model’s construction based on Pin1-bound references and its alignment with all characteristics defined in the constructed pharmacophore, all 658 retrieved molecules possess the requisite attributes essential for binding to Pin1. These 658 hit molecules were subsequently subjected to further *in silico* screening to identify the most promising candidates for subsequent investigation.

### 3.2 Docking and MM-GBSA calculations

The Docking is a valuable computational method employed for the discovery of novel Pin1 inhibitors, commonly utilized when there is available structural information concerning the target receptor (Ripphausen et al., 2012; Irwin and Shoichet, 2016). It facilitates the prediction of energetically favorable binding orientations of ligands to the target protein, enabling the prioritization of compound libraries based on their potential for effective interactions with the target (Tuccinardi et al., 2016). In this investigation, we conducted an *in silico* screening to identify molecules with superior docking scores compared to the reference ligand. We utilized the 658 hit molecules obtained from the Phase screening for this screening process. The *in silico* screening procedure consisted of two phases. Initially, the hits molecules underwent SP docking, resulting in the identification of 48 molecules with docking scores below −7.00 kcal/mol. These 48 molecules proceeded to the subsequent phase, which involved XP docking. From the XP docking outcomes, we identified 30 hit molecules with higher docking scores than the reference ligand.

To assess the binding stability of these 30 hit molecules with the Pin1 protein, we introduced a thermodynamic concept. We employed Prime MM/GBSA to filter and analyze the receptor-ligand binding modes generated by the XP docking simulation. This analysis aimed to ascertain the free binding energy and evaluate the binding stability of the 30 hit molecules. Our findings revealed that three molecules (SN0021307, SN0449787 and SN0079231) exhibited lower free binding energies (−57.12, −49.81 and −46.05 kcal/mol, respectively) than the reference ligand (−37.75 kcal/mol). Consequently, we eliminated the remaining hits, and the top three molecules with the lowest binding energies underwent interaction analysis.

Pin1 is a protein that can be subdivided into two distinct structural domains. The first domain is the catalytic PPIase domain, spanning residues 45 to 163. This domain is responsible for the arotamase function, which entails peptide-bond isomerization between proline and other amino acids. The second domain is the N-terminal WW domain, covering residues 1 to 39 (Ranganathan et al., 1997; Guo et al., 2009). The active pocket of the PPIase domain is the region where catalytic activity occurs, composed of specific amino acid residues including Lys63, Arg68, Arg69, Cys113, Leu122, Met130, Gln131, Phe134, Thr152, and Ser154. These residues engage with substrates and contribute to Pin1’s enzymatic activity (Guo et al., 2009).

**FIGURE 3:**
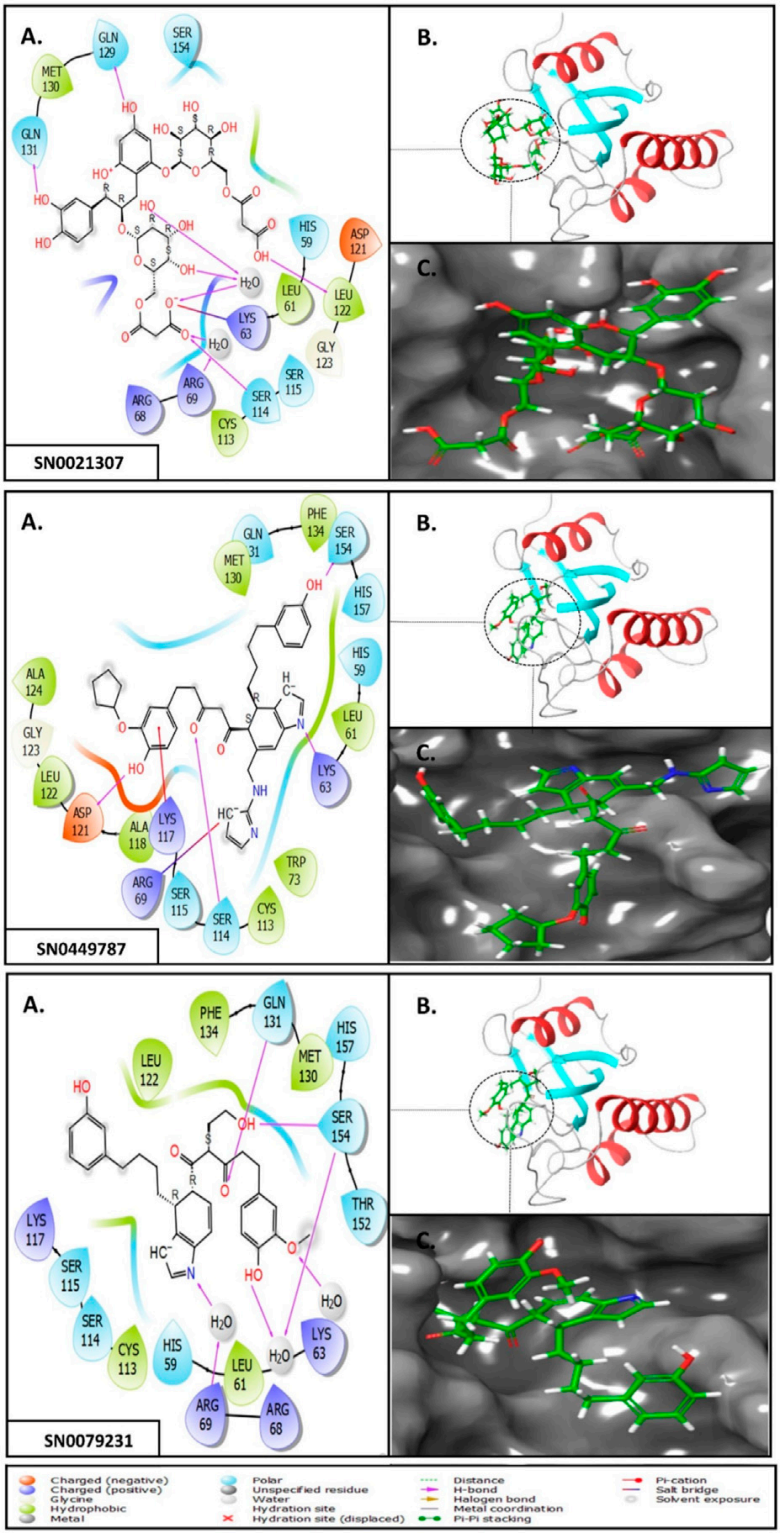
**(A)** 3D interaction diagrams; **(B)** Position of compounds in the Pin1 protein cavities; **(C)** 2D interaction diagrams.

To elucidate the structural binding mechanisms of the top three compounds to Pin1, we employed Maestro’s ligand-interaction diagram tool. This tool enabled us to show the docked conformations and interactions of these molecules with the crucial active site residues of Pin1, as illustrated in Figure 3. As a reference for our docking study, we selected the native ligand from the crystal structure of the Pin1 protein available in the PDB. The docking interaction analysis revealed that the hits possess the capability to bind to the active pocket within Pin1’s PPIase domain, as summarized in Table 1. These interactions encompassed the formation of hydrogen bonds with key residues, including LYS60, SER114, LEU122, GLN129, GLN131, ASP121, and ARG69. These findings are in concurrence with analogous studies in the literature, where other research teams have reported hydrogen bond interactions between their ligands and Gln131, Lys63, SER154, and ARG69. For instance, Zhang et al. identified hydrogen bond interactions between their ligands and Gln131 and Lys63, while Poli et al. and Ma et al. observed H-bond interactions with SER154 and ARG69 in their *in silico* investigations of Pin1 (Ma et al., 2019; Poli et al., 2022; Zhang et al., 2023).

**TABLE 1:** XP docking score, MM-GBSA, and the interaction of the top three compounds with Pin1 protein.

SN3 ID	2D structure	Docking score (kcal/mol)	MM-GBSA dG bind (kcal/mol)	Hydrogen bonding	Hydrophobic interactions
SN0021307	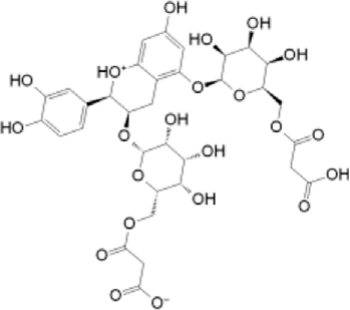	−9.891	−57.12	LYS60, SER114, LEU122, GLN129, GLN131	MET130, LEU61, LEU122, CYS113
SN0449787	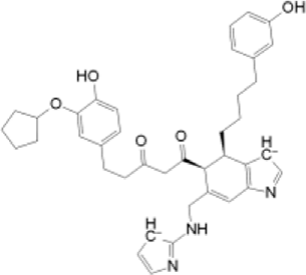	−7.579	−49.81	ASP121, SER154, LYS63, ARG69, SER114	PHE134, MET130, LEU61, ALA124, LEU122, ALA118, CYS113, TRP73
SN0079231	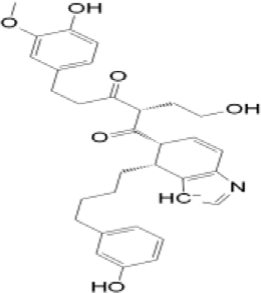	−7.097	−46.05	SER154, GLN131	MET130, PHE134, LEU61, CYS113, LEU122
Reference	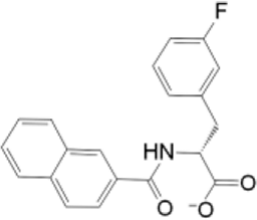	−6.064	−37.57	ARG69, LYS63, SER114	MET130, PHE134, LEU122, CYS113, LEU61

**FIGURE 4:**
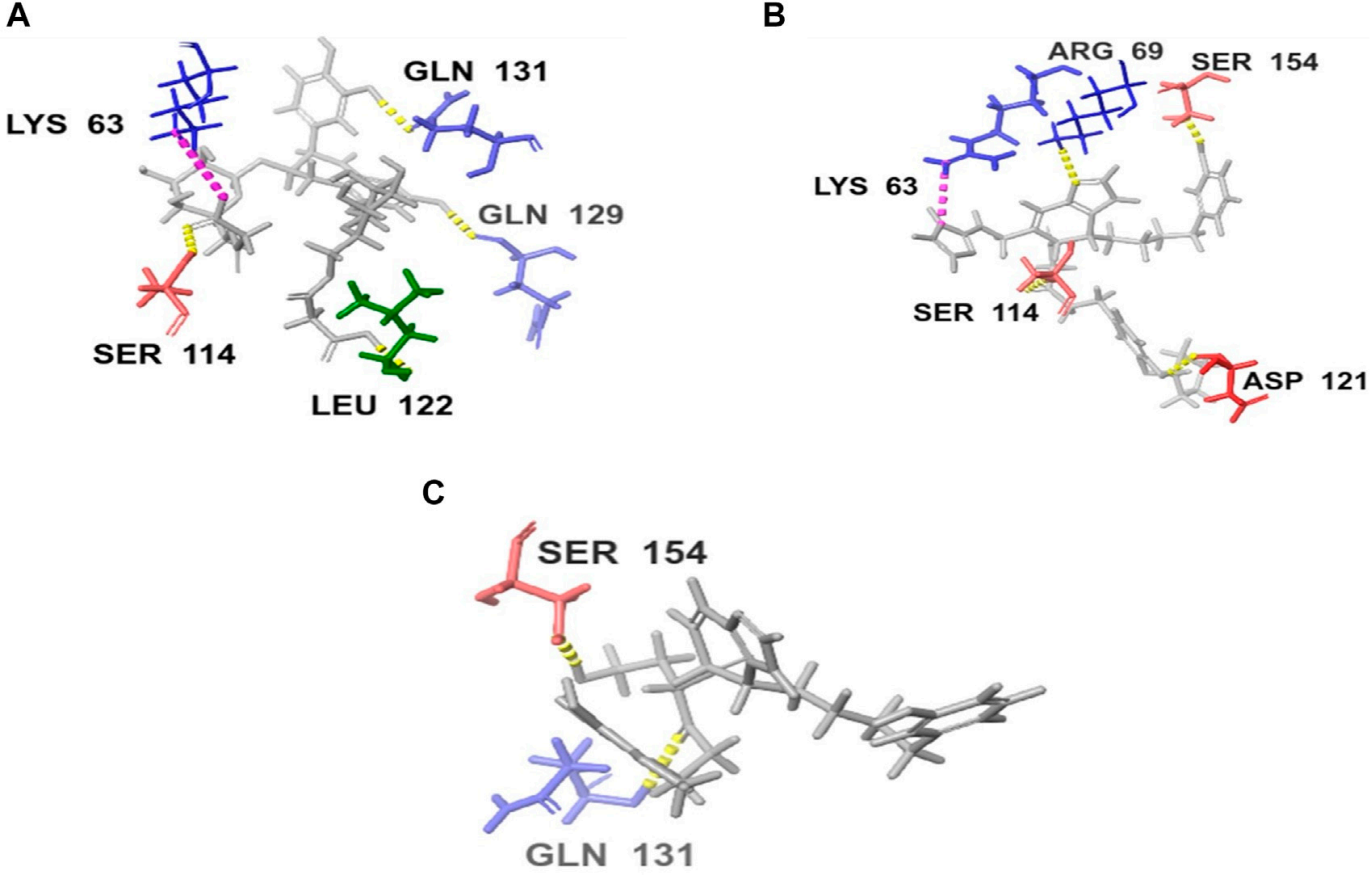
H-bond interaction of the top three compounds: **(A)** SN0021307; **(B)** SN0449787; **(C)** SN0079231.

Upon comparing the top molecules with the reference ligand, we observed shared hydrogen bond interactions with ARG69, LYS63, and SER114. Given that the formation of hydrogen bonds plays a pivotal role in determining the binding strength of ligands to proteins, the presence of one or more hydrogen bonds with key residues within the Pin1 domain is a critical factor in the design of new inhibitors. We conducted a comprehensive analysis of the hydrogen bonds (Figure 4), considering their quantity and distance, as presented in Table 2.

**TABLE 2:** H-bond analysis of the top three compounds with Pin1 protein.

Compound	Hydrogen bonding			
	No of H-bonds	Receptor residue	Ligand group	Distance (Å)
SN0021307	5	LYS63: NH2-	-O-	4.05
		SER114: NH-	-O=C-	2.34
		LEU122: C=O-	-OH	1.77
		GLN129: O-	-OH	1.67
		GLN131: C=O-	-OH	1.78
SN0449787	5	ASP121: C=O-	-OH	1.85
		SER154: O-	-OH	1.79
		LYS63: NH-	-N-	2.15
		ARG69: NH2-	-CH-	3.65
		SER114: OH-	-O=C-	2.37
SN0079231	2	Ser154: C=O-	-OH	1.71
		GLN131: NH-	-O=C-	2.08

Moreover, the hit molecules exhibited hydrophobic interactions with crucial residues, including PHE134, MET130, LEU61, LEU122, ALA118, CYS113, TRP73, and ALA124. Interestingly, the reference ligand exclusively manifested hydrophobic interactions with MET130, PHE134, LEU122, CYS113, and LEU61 residues, as outlined in Table 1. These findings harmonize with results from other research groups, such as Liu et al., who observed hydrophobic interactions involving Leu122, Cys113, and Ser114 in their ligand, and Poli et al., who reported interactions with LEU122 and MET130 (Liu et al., 2017; Poli et al., 2022).

Based on the docking scores and the comparative analysis of interactions with the reference and existing literature, the top three compounds were subjected to MD simulations.

### 3.3 Molecular dynamics simulations

Molecular dynamics (MD) is a computational tool that complements docking by enabling the study of protein-ligand complexes’ dynamic behavior over time. It plays a pivotal role in refining and validating docking predictions by assessing the reliability of predicted binding modes and filtering out compounds that fail to maintain stable interactions. MD simulations also yield diverse protein conformations for subsequent docking studies, thereby capturing the dynamic nature of the complex. These simulations consider various factors, including temperature and solvent effects, to provide insights into stability, flexibility, and conformational changes (Aghazadeh Tabrizi et al., 2016; Offutt et al., 2016; Bharatham et al., 2017).

In this study, we confirmed the conformational stability and interactions observed during MD simulations of the Pin1 protein with the top three compounds (SN0021307, SN0449787 and SN0079231) and its native ligand through several analyses, including RMSD Analysis, RMSF Analysis, Interaction Analysis, and Ligand Stability Analysis.

**FIGURE 5:**
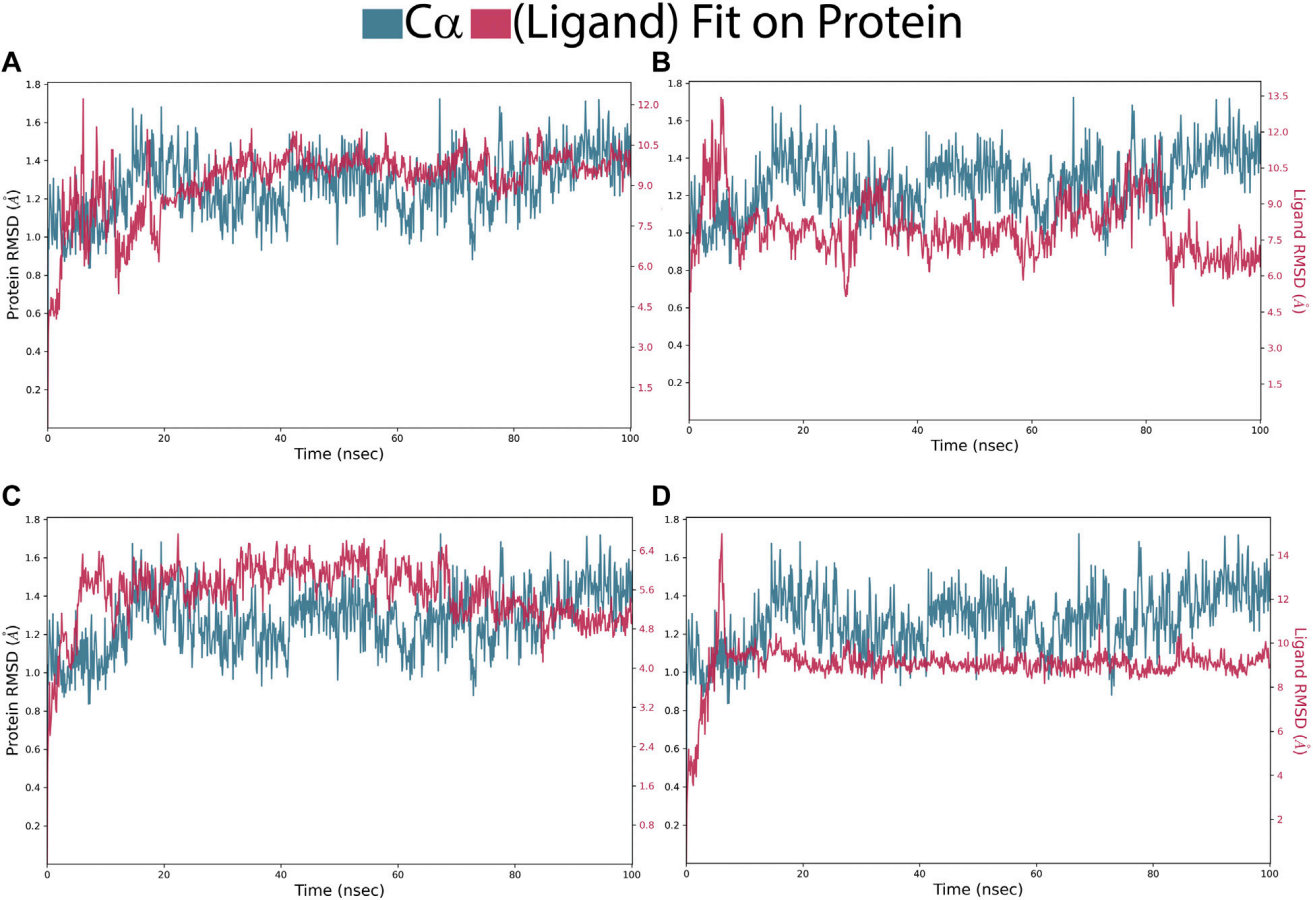
RMSD of Pin1-complexes: **(A)** SN0021307; **(B)** SN0449787; **(C)** SN0079231 **(D)**; Reference.

In the Pin1-SN0021307 complex, the receptor exhibited slight fluctuations ranging between 0.8 and 1.6 Å in the first 20 ns but then stabilized with an RMSD between 1.2 and 1.6 Å throughout the simulation (Figure 5; Table 3). The ligand initially showed fluctuations with an RMSD ranging from 0.6 to 1.8 Å but eventually stabilized, maintaining strong interactions with the protein for the remainder of the simulation. In the Pin1-SN0449787 complex, the receptor displayed fluctuations with an RMSD range of 0.8–1.6 Å in the first 20 ns, followed by equilibration with fluctuations between 1.2 and 1.6 Å. The ligand exhibited fluctuations at specific time intervals but overall remained stable and strongly interacted with the receptor during most of the simulation. In contrast, in the Pin1-SN0079231 complex, the receptor remained stable with slight deviations and an RMSD value ranging from 1 to 1.6 Å, while the ligand maintained equilibrium with slight fluctuations with the protein throughout the simulation. Regarding the bound ligand, the protein remained stable with minimal fluctuations, except for the initial 5 ns. The ligand also maintained equilibrium with slight deviations and consistent contact with the protein. These RMSD analyses collectively suggest that the studied compounds and the native ligand form stable complexes with Pin1.

**TABLE 3:** Average of RMSD and RMSF values for the top two compounds and the reference ligands.

Compound	RMSD		RMSF	
	Cα (Å)	ligand (Å)	Cα (Å)	ligand (Å)
SN0021307	1.27 ± 0.16	2.93 ± 0.29	0.72 ± 0.37	1.4 ± 0.77
SN0449787	1.27 ± 0.16	3.08 ± 0.57	0.72 ± 0.37	2.33 ± 1.09
SN0079231	1.27 ± 0.16	3.5 ± 0.52	0.72 ± 0.37	0.89 ± 0.39
Reference	2.54 ± 0.39	1.75 ± 0.27	0.72 ± 0.37	1.06 ± 0.41

**FIGURE 6:**
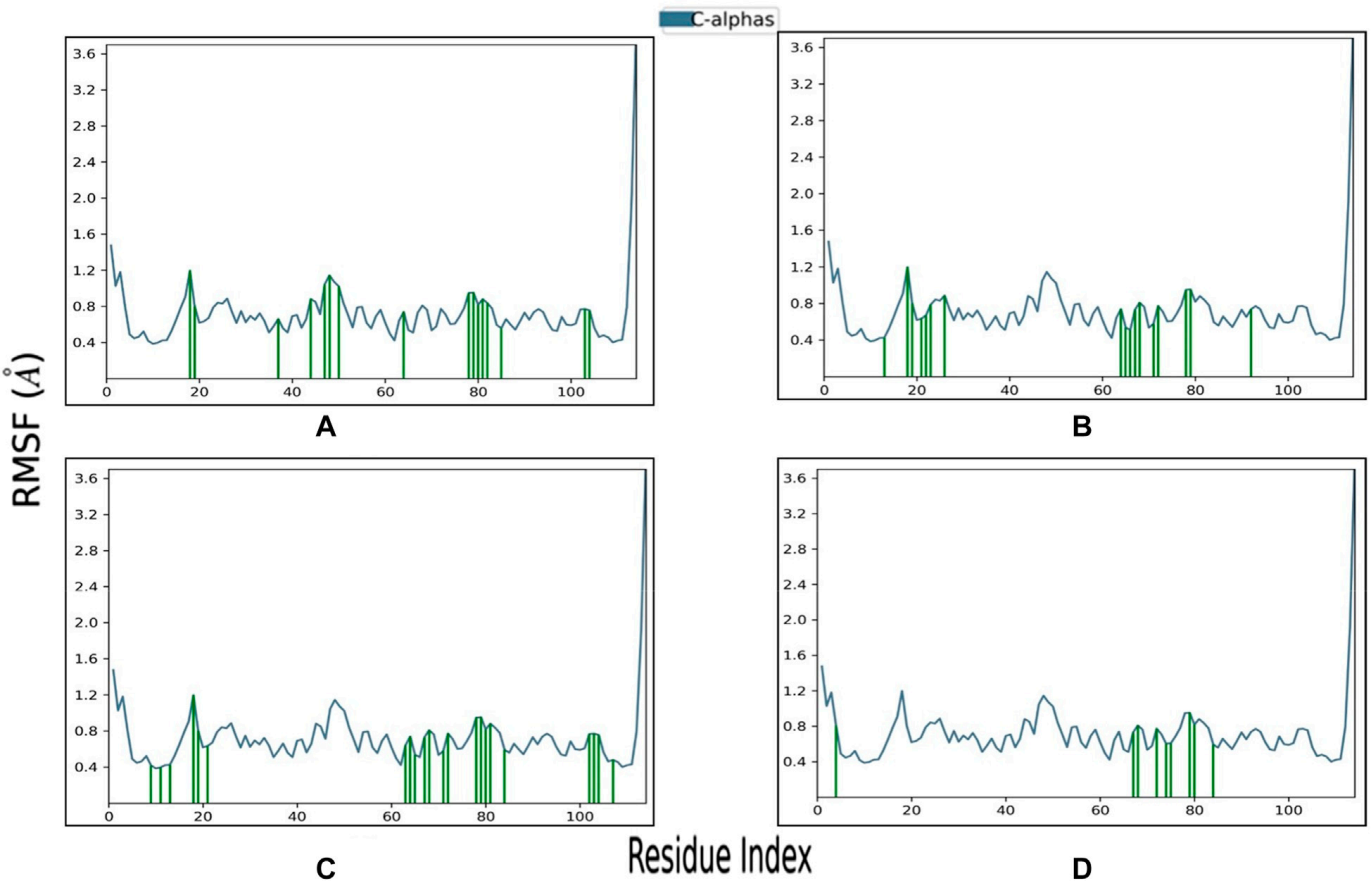
RMSF profile of Pin1 protein with the three complexes: **(A)** SN0021307; **(B)** SN0449787; **(C)** SN0079231; **(D)** Reference.

Root Mean Square Fluctuation (RMSF) is a valuable property for assessing the structural flexibility of proteins during MD. It provides insights into the fluctuations of specific amino acid residues and helps evaluate the impact of ligand binding on protein stability. In this study, we calculated relative RMSF values to analyze the fluctuations of amino acid residues within the protein when bound to the ligands. An RMSF value exceeding 3 Å suggests increased amino acid fluctuations upon ligand binding, indicating potential disruption of the protein’s structural stability. Conversely, an RMSF value below 3 Å implies reduced amino acid fluctuations, indicating enhanced protein stability and resistance to structural changes. For the selected complexes in this study, RMSF values ranged from 0.4 to 3 Å, reflecting moderate fluctuations in amino acid residues (Figure 6; Table 3). The average RMSF values of the protein bound the ligands as follow: 1.4 Å for SN0449787, 2.33 Å for SN0021307, 0.89 Å for SN0079231, and 1.06 Å for the reference ligand. These RMSF values collectively indicate both the stability and flexibility of the complexes during the MD simulations.

**FIGURE 7:**
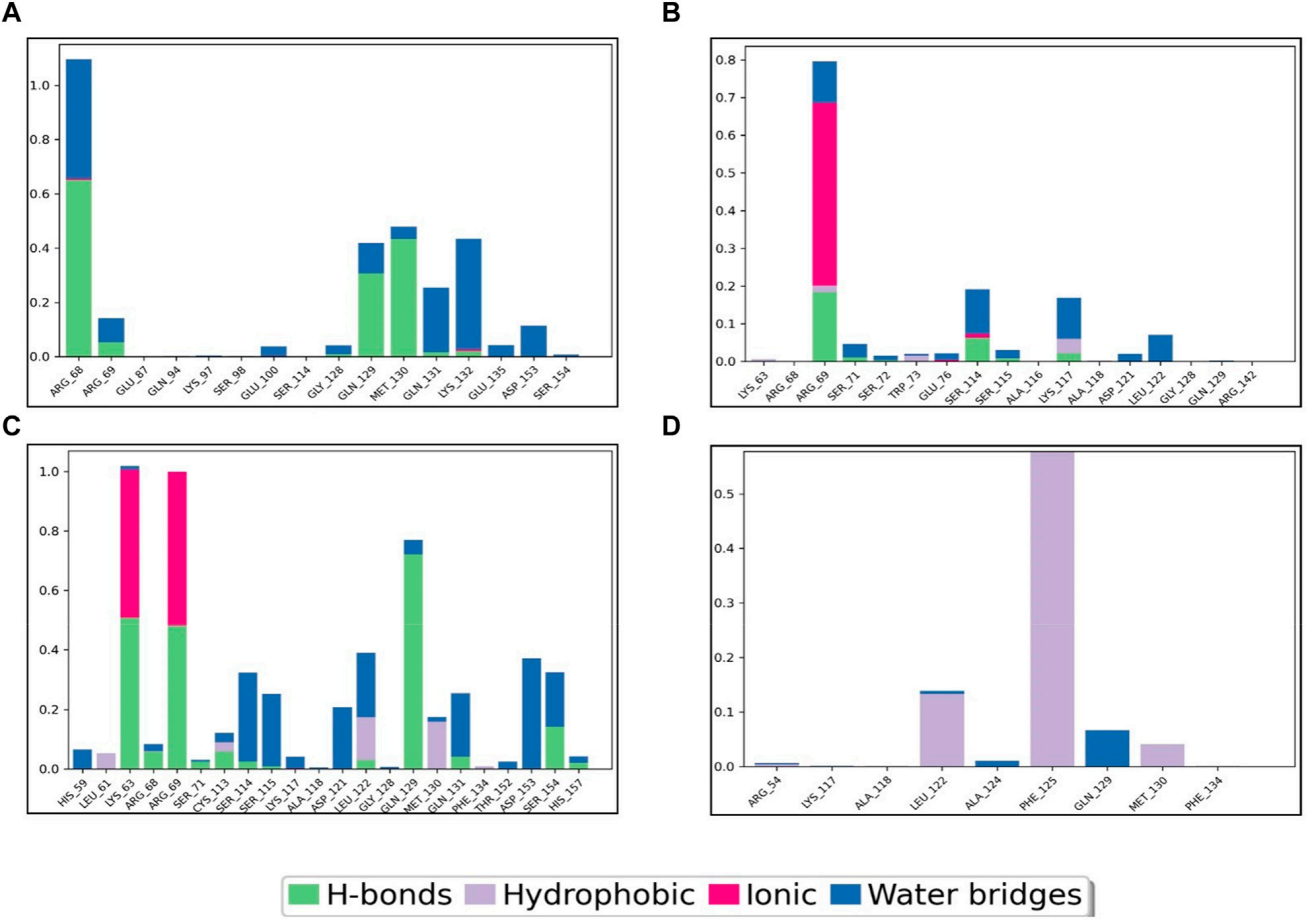
Pin1-complex’s interaction diagram: **(A)** SN0021307; **(B)** SN0449787; **(C)** SN0079231; **(D)** Reference.

To gain deeper insights into the potential of the designed molecules as Pin1 inhibitors, we analyzed the interactions between the molecules and key residues within the binding site during the 100 ns MD simulations. Figure 7 provides a schematic representation of these interactions, including their formation percentages. These interactions were categorized into various types, including direct hydrogen bonds, indirect hydrogen bonds via water bridges, as well as hydrophobic and ionic interactions. The specific residues involved in these interactions were identified. The formation percentages allow us to assess the stability and frequency of these interactions throughout the simulation. By analyzing these percentages, we can identify compounds that consistently form favorable interactions with Pin1, indicating their potential as effective inhibitors.

For instance, SN0449787 demonstrated both direct and indirect hydrogen bond interactions with several key residues within the Pin1 binding site. Direct hydrogen bonds were formed with ARG68 (65%), MET130 (45%), and GLN129 (40%), among others. Indirect interactions, mediated by water bridges, were observed with residues such as LYS132, ARG68, ARG69, GLN129, MET130, GLN131, GLU135, ASP153, SER154, and GLU100. Ionic interactions were noted with ARG68 and LYS132. The interactions of the other complexes are detailed in Table 4, with the reference ligand showing no direct hydrogen bond interactions.

**TABLE 4:** Interactions percentage of the top three compounds and the reference ligand.

Compound	HB	Hydrophobic	Water bridge	Ionic
SN0021307	ARG68(65%), MET130(45%), GLN129(40%), ARG69, GLY128, GLN131, LYS132	—	LYS132(40%), ARG68(35%), ARG69, GLN129, MET130, GLN131, GLU135, ASP153, SER154, GLU 100	ARG68, LYS132
SN0449787	ARG69, SER71, SER114, LYS117	ARG69, TRP73, LYS117	ARG69, SER71, GLU76, SER1115, LYS117, ASP121, LEU122	ARG69 (50%)
SN0079231	GLN129(70%), LYS63(40%), ARG69(40%), SER154, ARG67, SER71, SER114, LEU122, GLN131	MET130, LEU122, LEU61, LYS113, PHE134	ASP153(40%), SER115, SER114, LYS117, ASP121, LEU122, GLN129, GLN131, SER154, HIS157, HIS59	LYS63(45%), ARG69(45%)
Reference	—	PHE125(60%), LEU122, MET139, ARG54	GLN129, ARG54, ALA124	—

**FIGURE 8:**
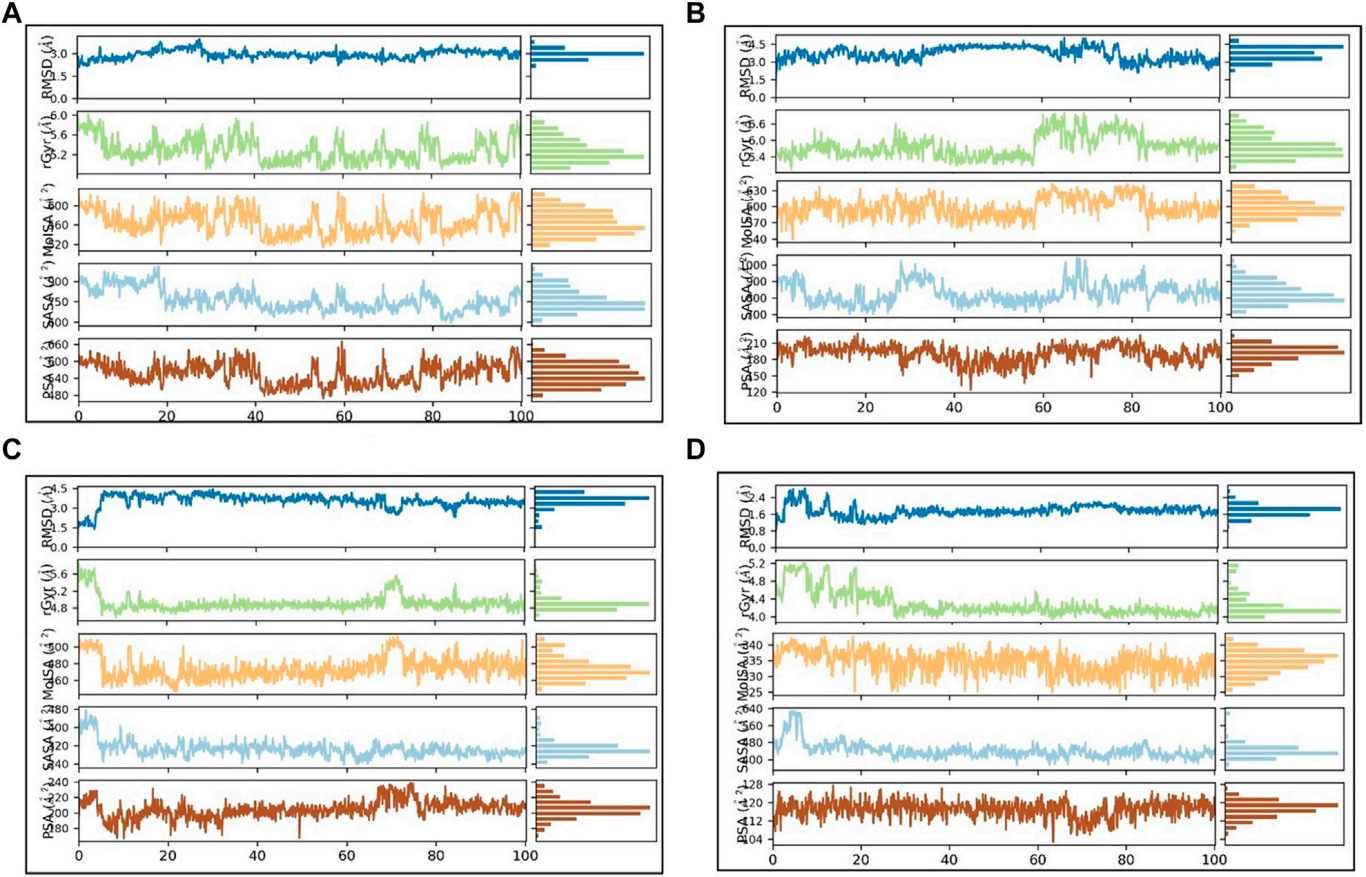
PL-contacts diagram: **(A)** SN0021307 **(B)** SN0449787 **(C)** SN0079231 **(D)** Reference.

The Ligand RMSD values for SN0021307, SN0449787, SN0079231, and the reference ligand spanned from 0.2 to 3 Å, 3 to 3.5 Å, 1.5 to 4 Å, and 0.8 to 2.4 Å, respectively (Figure 8; Table 5). Their respective average values were 1.38 Å, 0.74 Å, 1.56 Å, and 0.63 Å. Additionally, the rGyr values, indicative of protein folding status, exhibited fluctuations between 5.2 and 6 Å for SN0021307, 5.4–6.6 Å for SN0449787, 4.8–5.6 Å for SN0079231, and 4–5.2 Å for the reference ligand. To assess the exposure of the protein complexes to solvent molecules and their structural stability, various surface area measurements, including MolSA, SASA, and PSA, were examined.

**TABLE 5:** Average rGyr and SASA values for the top compounds and the reference ligands.

Compound	RMSD (Å)	rGyr (Å)	MolSA (Å)	SASA (Å)	PSA (Å)
SN0021307	2.93 ± 0.29	5.28 ± 0.24	565.5 ± 25.7^2^	762.9 ± 79.4^2^	555.2 ± 38.1^2^
SN0449787	3.08 ± 0.57	5.78 ± 0.4	598.5 ± 18.8^2^	828.9 ± 61.9^2^	190.9 ± 15.6^2^
SN0079231	3.5 ± 0.52	4.9 ± 0.19	474.1 ± 13.1^2^	304.9 ± 32.6^2^	204.9 ± 11.4^2^
Reference	1.75 ± 0.27	4.2 ± 0.28	334.4 ± 3.5^2^	441.7 ± 38.7^2^	117.1 ± 3.5^2^

For SN0021307, MolSA ranged from 520–600 Å^2^, SASA from 600–900 Å^2^, and PSA from 540–600 Å^2^, with average values estimated at 565.5 Å^2^, 762.9 Å^2^, and 555.2 Å^2^, respectively. In the case of SN0449787, MolSA spanned 570–630 Å^2^, SASA from 700–900 Å^2^, and PSA from 150–210 Å^2^, with average values of 598.5 Å^2^, 828.9 Å^2^, and 190.9 Å^2^, respectively. SN0079231 exhibited MolSA between 460 and 590 Å^2^, SASA from 420–480 Å^2^, and PSA from 180–240 Å^2^, with average values of 474.1 Å^2^, 304.9 Å^2^, and 204.9 Å^2^, respectively. In the case of the reference, MolSA, SASA, and PSA ranged from 330–340 Å^2^, 400–560 Å^2^, and 104–128 Å^2^, respectively, with average values of 334.4 Å^2^, 441.7 Å^2^, and 117.1 Å^2^. These findings indicate that the values for each ligand remained within specific ranges, signifying stability and successful complex formation.

The utilization of Computer-Aided Drug Design (CADD) tools in drug discovery research has established new standards for identifying promising chemical compounds targeting specific receptors. It is undeniable that CADD approaches significantly contribute to enhancing the drug discovery pipeline. The four proposed molecules exhibit promising binding affinity towards PIN1, as evidenced by molecular docking, MM-GBSA calculations, and MD simulation studies. However, to validate the potential of these molecules against PIN1, several experimental verifications are imperative. The Surface Plasmon Resonance (SPR) assay presents an effective method for confirming the binding affinity of the molecules. A kinetic study offers a suitable approach to scrutinize the binding and unbinding mechanisms of these molecules. Further optimization of the molecules may be necessary based on experimental assessments to improve their efficacy and therapeutic potential.

## 4 Conclusion

This research focused on targeting the distinctive enzyme PIN1, crucial for catalyzing proline isomerization within phospho-serine/threonine-proline motifs, with significant therapeutic implications for cancer treatment. Employing an *in silico* approach, the study utilized an e-pharmacophore model to guide the screening of a library of natural products (NPs) from the SN3 database. The screening process was refined through SP and XP docking filters, yielding 34 lead compounds with superior binding affinity compared to the co-crystalized inhibitor. Further refinement employed Prime/MM-GBSA calculations, identifying seven compounds with higher binding energies than the reference ligand. To validate their potential as PIN1 inhibitors and assess stability, molecular dynamics (MD) simulations were conducted, confirming stable binding interactions with the PIN1 protein for the identified lead compounds. Notably, three phytochemicals—SN0021307, SN0449787, and SN0079231—emerged with remarkable affinity toward the PIN1 protein.

## Data Availability

The raw data supporting the conclusion of this article will be made available by the authors, without undue reservation.

## Abbreviations

CPH, common pharmacophoric hypothesis; EMM, molecular mechanics energy; HB, Hydrogen bonds; IARC, International Agency for Research on Cancer; MD, Molecular Dynamics; MM-GBSA, Molecular mechanics with generalized Born and surface area; MolSA, molecular surface area; NP, natural product; NPT, isothermal–isobaric ensemble; Pin1, protein interacting with never-in-mitosis akinase-1; PSA, Vander waal surface area of polar nitrogen and oxygen; PPW, protein preparation wizard; Rg, Radius of Gyration; RMSD, Root Mean Square Deviation; RMSF, Root Mean Square Fluctuation; SASA, solvent accessible surface area; SGBM, surface generalized Born model; SN3, supernatural 3.0; SP, Standard Precision; SPME, Smooth Particle Mesh Ewald; VSGB, polar solvation energy; XP, Extra precision.
